# Daily rhythms influence the ability of lung-derived extracellular vesicles to modulate bone marrow cell phenotype

**DOI:** 10.1371/journal.pone.0207444

**Published:** 2018-11-26

**Authors:** Mark S. Dooner, Connor Stewart, Yanhui Deng, Elaine Papa, Mandy Pereira, Michael Del Tatto, Shannon Johnson, Sicheng Wen, Ashley Amaral, Jason Aliotta, Peter J. Quesenberry, Laura R. Goldberg

**Affiliations:** Department of Medicine, Division of Hematology/Oncology, Rhode Island Hospital, Warren Alpert Medical School of Brown University, Providence, RI, United States of America; McGill University, CANADA

## Abstract

Extracellular vesicles (EVs) are important mediators of intercellular communication and have been implicated in myriad physiologic and pathologic processes within the hematopoietic system. Numerous factors influence the ability of EVs to communicate with target marrow cells, but little is known about how circadian oscillations alter EV function. In order to explore the effects of daily rhythms on EV-mediated intercellular communication, we used a well-established model of lung-derived EV modulation of the marrow cell transcriptome. In this model, co-culture of whole bone marrow cells (WBM) with lung-derived EVs induces expression of pulmonary specific mRNAs in the target WBM. To determine if daily rhythms play a role in this phenotype modulation, C57BL/6 mice were entrained in 12-hour light/12-hour dark boxes. Lungs harvested at discrete time-points throughout the 24-hour cycle were co-cultured across a cell-impermeable membrane with murine WBM. Alternatively, WBM harvested at discrete time-points was co-cultured with lung-derived EVs. Target WBM was collected 24hrs after co-culture and analyzed for the presence of pulmonary specific mRNA levels by RT-PCR. In both cases, there were clear time-dependent variations in the patterns of pulmonary specific mRNA levels when either the daily time-point of the lung donor or the daily time-point of the recipient marrow cells was altered. In general, WBM had peak pulmonary-specific mRNA levels when exposed to lung harvested at Zeitgeber time (ZT) 4 and ZT 16 (ZT 0 defined as the time of lights on, ZT 12 defined as the time of lights off), and was most susceptible to lung-derived EV modulation when target marrow itself was harvested at ZT 8- ZT 12. We found increased uptake of EVs when the time-point of the receptor WBM was between ZT 20 -ZT 24, suggesting that the time of day-dependent changes in transcriptome modulation by the EVs were not due simply to differential EV uptake. Based on these data, we conclude that circadian rhythms can modulate EV-mediated intercellular communication.

## Introduction

EVs, membrane-bound cellular particles containing DNA, RNA, lipids and protein, are key players in inter-cellular communication [1–4]. They have been implicated in numerous physiologic and pathophysiologic processes, have been shown to have potent tissue restorative properties, and are being extensively studied as biomarkers for many disease states [5–10]. Strategies to harness their therapeutic potential and inhibit their disease-causing effects have tremendous clinical applicability. Numerous factors influence EV biogenesis, trafficking and function [11–13]. Circadian rhythm is a fundamental regulator of innumerable biologic processes [14–23] and as such, it is likely playing an important albeit, currently poorly understood, role in EV function. In support of this, circadian variation in VCAM-1 positive EVs and tissue-factor positive EVs in human subjects has been documented [24]. Danielson et al, utilizing nano flow cytometry, also found diurnal variations in the size distribution of peripheral blood-derived EVs in 6 adults, with a trend toward larger EVs and largest range of EV sizes in the evening compared to the morning [25]. Elegant studies have shown circadian-related modulation of circulating microRNAs targeting the clock genes [26], and diurnal variation was also found in urinary exosomal NaCl cotransporter and prostasin (decreased in am, increased in the pm) [27]. However, to date, very few studies have examined the role of circadian rhythm in the ability of EVs to functionally modulate target cell phenotype. In these studies, to dissect out the role of daily rhythms in EV-mediated communication, we employed a well-established system in which lung-derived EVs, when co-cultured with marrow stem cells, induce the marrow cells to express pulmonary epithelial cell-specific mRNA and protein [28–29]. Using this established, easily manipulated in vitro system, we determined how time of day influences the ability of lung-derived EVs to affect phenotype change in target bone marrow cells.

## Materials and methods

### Mice

Male C57BL/6 mice were purchased from Jackson Laboratory (Bar Harbour, ME), 6–8 weeks old and maintained in light/dark cabinets on a 12-hour light/12-hour dark schedule for 2 weeks prior to experiments. They were housed in a clean facility in accordance with the National Research Council’s Guide for the Care and Use of Laboratory Animals [National Research Council (1996) Guide for the care and use of laboratory animals. Washington, DC: National Academy Press] and were given food and water ad libitum. All experiments were approved by the Rhode Island Hospital Institutional Animal Care and Use Committee.

### Quantification of EVs from lung, blood and marrow cells at different time-points throughout a 12-hour light/12-hour dark cycle

C57BL/6 mice were entrained in 12-hour light/12-hour dark boxes for two weeks. At discrete Zeitgeber times (ZT), with ZT 0 defined as the time of lights turned on (7am) and ZT 12 defined as the time of lights turned off (7pm), mice were sacrificed by isoflurane inhalation followed by cervical dislocation in accordance with the National Research Council’s Guide for the Care and Use of Laboratory Animals. Blood, lung and marrow cells were harvested. EVs were obtained from blood as follows: blood was centrifuged at 1900g for 10 minutes (min), supernatant was recovered and centrifuged at 5000g for 20 min, supernatant was recovered and centrifuged at 100,000g for 70 min, the pellet was collected, washed, and centrifuged at 100,000g for 70 min. The final EV pellet was re-suspended in PBS for analysis. For marrow, femora, tibiae and iliac crests were flushed using a 22.5 gauge needle in 1X phosphate-buffered saline (PBS). The cells were centrifuged at 300g for 20 min and both the cellular pellet and the supernatant were recovered. The cell pellet was resuspended in PBS and the number of marrow cells was counted. The supernatant was recovered and centrifuged at 2000g for 20 min, supernatant again was recovered and centrifuged at 100,000g for 70 min. The resulting pellet was recovered, re-suspended in PBS and centrifuged at 100,000g for 70 min. This final pellet was re-suspended in PBS for EV analysis. For lung, lung tissue was dissociated in PBS/2.5units/mL dispase/0.01% DNase for 45 min, then in DMEM/5% EV-depleted FBS/0.01% DNase for 20 min, then centrifuged at 300g for 10 min, supernatant was recovered and centrifuged at 2000g for 20 min, supernatant was recovered and centrifuged at 100,000g for 70 min, the pellet was recovered, re-suspended in PBS and centrifuged at 100,000g for 70 min. The final pellet was re-suspended in PBS for analysis. EVs were quantified using Nanoparticle Tracking Analysis (Malvern Instruments, UK) per manufacturer’s protocol. The number of EV particles was normalized to mg of input lung tissue, number of marrow cells, and mL of input blood for lung-derived, marrow-derived, and blood-derived EVs respectively.

### Assessing effects of changing the time of day of the harvest of EV donor tissue on the ability of the EVs to mediate phenotype changes in target WBM

At discrete ZTs, lungs were harvested from entrained C57BL/6 mice, mechanically minced and co-cultured with WBM (all harvested from mice consistently at ZT 9) in DMEM/15% EV-depleted FBS/1% penicillin/1% streptomycin/50ng/mL stem cell factor. Lung was separated from WBM by a 0.4uM cell-impermeable well insert. After 24 hours, WBM was collected and RNA was isolated from the WBM and used to amplify cDNA (High Capacity cDNA Reverse Transcription Kit, Applied Biosystems). Gene expression for 6 pulmonary epithelial genes-surfactant A (Mm00499170_m1), surfactant B (Mm00455681_m1), surfactant C (Mm00488144_m1), clara cell specific protein (Mm00442406_M1), and aquaporin 5 (Mm00437578_m1)- was analyzed by RT-PCR (9800 Fast Thermal Cycler, Applied Biosystems). The primers were purchased from Applied Biosystems. They were not validated in our own laboratory. All 20X assay mixes were purchase from Applied Biosystems. The mean cycle threshold (CT) value of the target gene in each sample was normalized to its housekeeping gene (β2 microglobulin; Mm00437762_M1) CT value to yield a ΔCT which was then normalized to the WBM alone control sample (ΔΔCT). There were no statistically significant differences in expression of the housekeeping gene, β2 microglobulin, between any of the samples, indicating no time of day-dependent changes in expression of this gene (data not shown). When comparing CT values for WBM alone (normalized to β2 microglobulin within each sample), there were no statistically significant differences in expression between time points for surfactant A, surfactant B, surfactant C and clara cell specific protein, but there was a slight increase in aquaporin 5 at ZT 24 vs. ZT 8 and ZT 24 vs. ZT 16 (p = 0.04, 0.05 respectively). However, the ΔCT for each sample was normalized to the WBM alone control and therefore, such normalization should serve as an internal control for these minor time of day dependent changes in WBM alone for aquaporin 5. The 2^-ΔΔCT^ (RQ value) was then calculated for each target gene. We interpreted a CT value = 35 as equivalent to no expression. Therefore, for target genes where no expression was found in the control group (defined as a CT value of greater than or equal to 35), a CT value of 35 was assigned so relative quantification of the target gene could be calculated. The CT values for the genes across all ZTs ranged between 26–33.7, 23–25, 25.6–33, 27–34, 21–31, and 27.6–30.8 for surfactant A, surfactant B, surfactant C, surfactant D, clara cell specific protein and aquaporin 5 respectively.

### Assessing effects of changing the WBM time of day in EV-mediated phenotype modulation

For lung-derived EV preparation, lungs were harvested from mice at ZT 2, minced, and cultured in bronchial EGM/10% EV-depleted FBS /1% penicillin/1% streptomycin. After 48 hours, conditioned media was collected, centrifuged at 300g for 10 min, supernatant was recovered and centrifuged at 100,000g for 70 min, the pellet was collected, washed in PBS, and centrifuged at 100,000g for 70 min to yield the final EV pellet. The pellet was resuspended in PBS/10%DMSO, subjected to protein quantification using the BCA assay and frozen in aliquots until use within 48 hours. At different ZTs, mice were sacrificed, WBM was flushed from bones, washed, and 20 million WBM cells were plated with 20μg EVs in DMEM/15%EV-depleted FBS/1% penicillin/1% streptomycin/50ng/mL stem cell factor. After 24 hours in co-culture, WBM was harvested, RNA isolated, and levels of pulmonary specific genes in WBM were analyzed by RT-PCR as described above.

### Assessing the influence of changing the time of day of target WBM on EV uptake

Lungs from 5 C57BL/6 mice were harvested at ZT 2, mechanically minced and placed in culture in DMEM/10%EV-depleted FBS/1% penicillin/1% streptomycin for 48 hours. Conditioned media 48 hours after culture was collected and the lung-derived EVs were isolated by differential centrifugation (300g for 10 min, supernatant 100,000g for 70 min, pellet recovered, washed and centrifuged 100,000g for 70 min, final pellet resuspended in PBS). The lung-derived EVs have been extensively characterized and described in detail [28–31]. These lung-derived EVs were labeled with DiI (Vybrant Cell-Invitrogen) per manufacturer’s protocol with the following changes: EVs were suspended in 1 mL serum-free phenol-red free medium (DMEM), 5 μL of Vybrant DiI V22885 was added to the EV suspension, incubated 20 min at 37°C, then washed and centrifuged at 100,000g for 70 min twice. The final pellet was resuspended in 500μL DMEM, and protein assay was done by BCA. DiI in the absence of EVs was processed in parallel and incubated with WBM to test for WBM labeling by DiI alone as an additional negative control. WBM was then harvested from mice at 6 different ZTs, 3 mice/time-point, and incubated with or without the DiI-labeled lung-derived EVs for 24 hours. Following co-culture, WBM was collected, washed and analyzed for EV-uptake by flow cytometry. Flow cytometry was performed on a FACScan (Becton Dickinson). Cells were gated using FSC/SSC, and then further gated for DiI-positivity. Analysis was done using FlowJo, LLC software. The percent DiI-positive cells for each ZT was calculated by subtracting the percent positive cells in the control sample (WBM alone) from that of the samples (WBM + EV). Negative control samples (WBM alone) contained <0.10% positive cells at every ZT.

### Statistical analysis

Data are presented as average RQ ± standard error of the mean. Non-parametric Wilcoxon rank-sum tests were used to test for significant differences between experimental groups. Pair-wise comparisons were made and we considered results to be statistically significant when p <0.05. All p-values are two-sided.

## Results

**Fig 1:**
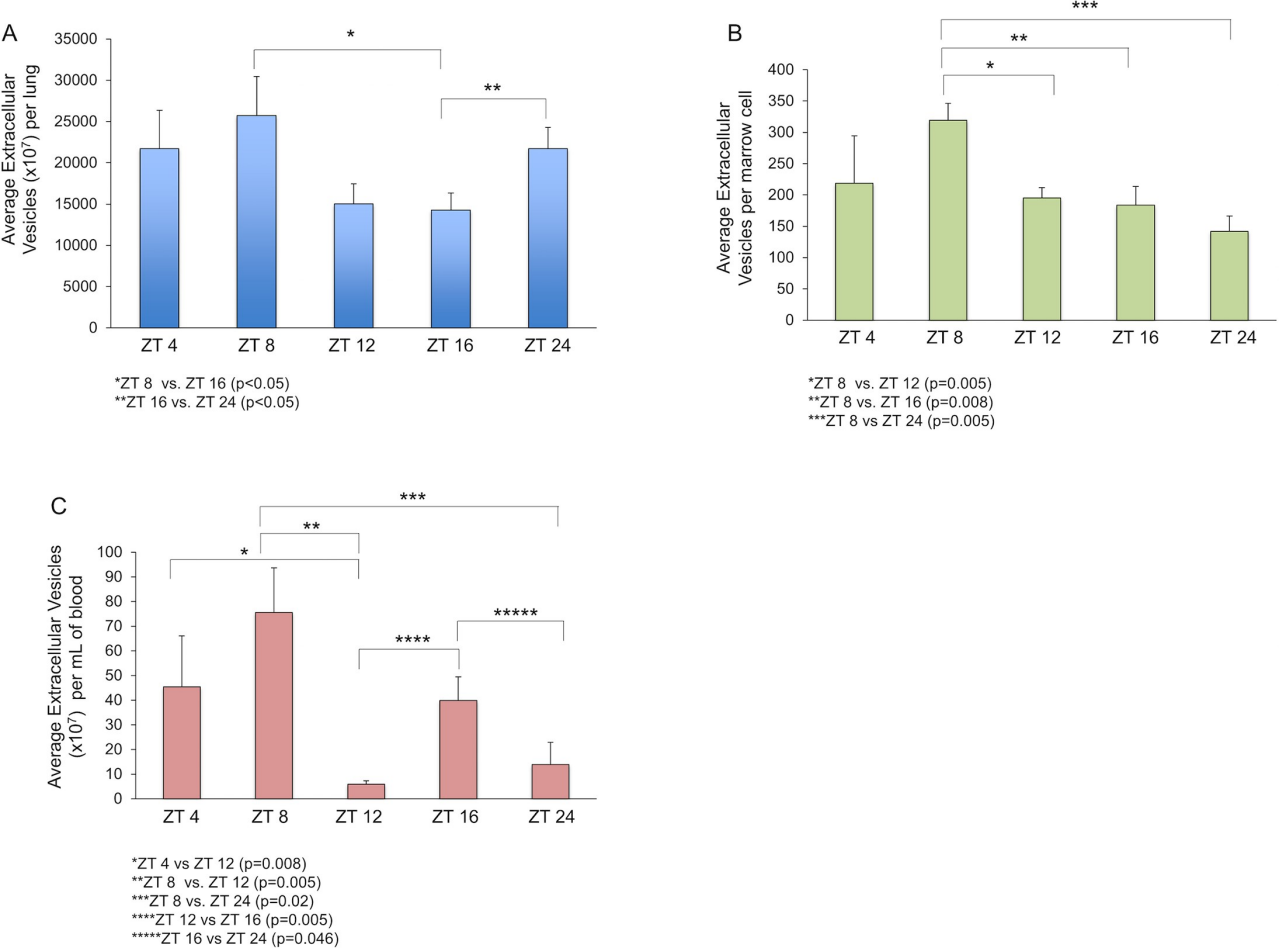
There were time of day-dependent statistically significant differences in EV quantity from murine lung tissue, bone marrow cells, and peripheral blood. C57BL/6 mice were entrained in 12-hour light/12-hour dark boxes for 2 weeks. At 5 discrete time-points- ZT 4, ZT 8, ZT 12, ZT 16, and ZT 24, mice were sacrificed and EVs from whole bone marrow cells, lung tissue, and peripheral blood were isolated and quantified using Nanoparticle Tracking Analysis per manufacturer’s protocol. The number of EV particles per murine lung (A), per marrow cell (B), and per mL of peripheral blood (C) are shown. Each bar represents the average number of EVs/tissue ± standard error of the mean from 1–2 separate experiments per ZT (total n = 5–9 mice/time-point). *p<0.05 or as specified in the Figure (Wilcoxon rank-sum test).

We determined if there were any time of day-related changes in the quantity of EVs able to be isolated from peripheral blood, marrow and lung. We entrained C57BL/6 mice for 2 weeks in 12-hour cycle light/dark boxes. After the two weeks, peripheral blood, bone marrow cells and lung were harvested from the entrained mice at five distinct ZTs. Extracellular vesicles (EVs) were isolated from each tissue using differential centrifugation and quantified using Nanoparticle Tracking Analysis. The number of EV particles was normalized to mg of input lung tissue, number of marrow cells, and mL of input blood for lung-derived, marrow-derived, and blood-derived EVs, respectively. As shown in Fig 1, there were time of day-dependent statistically significant differences in EV quantity from all three tissues. Peak EV quantity was seen at ZT 8 for all three tissues. There were additional tissue-specific peaks and nadirs.

**Fig 2:**
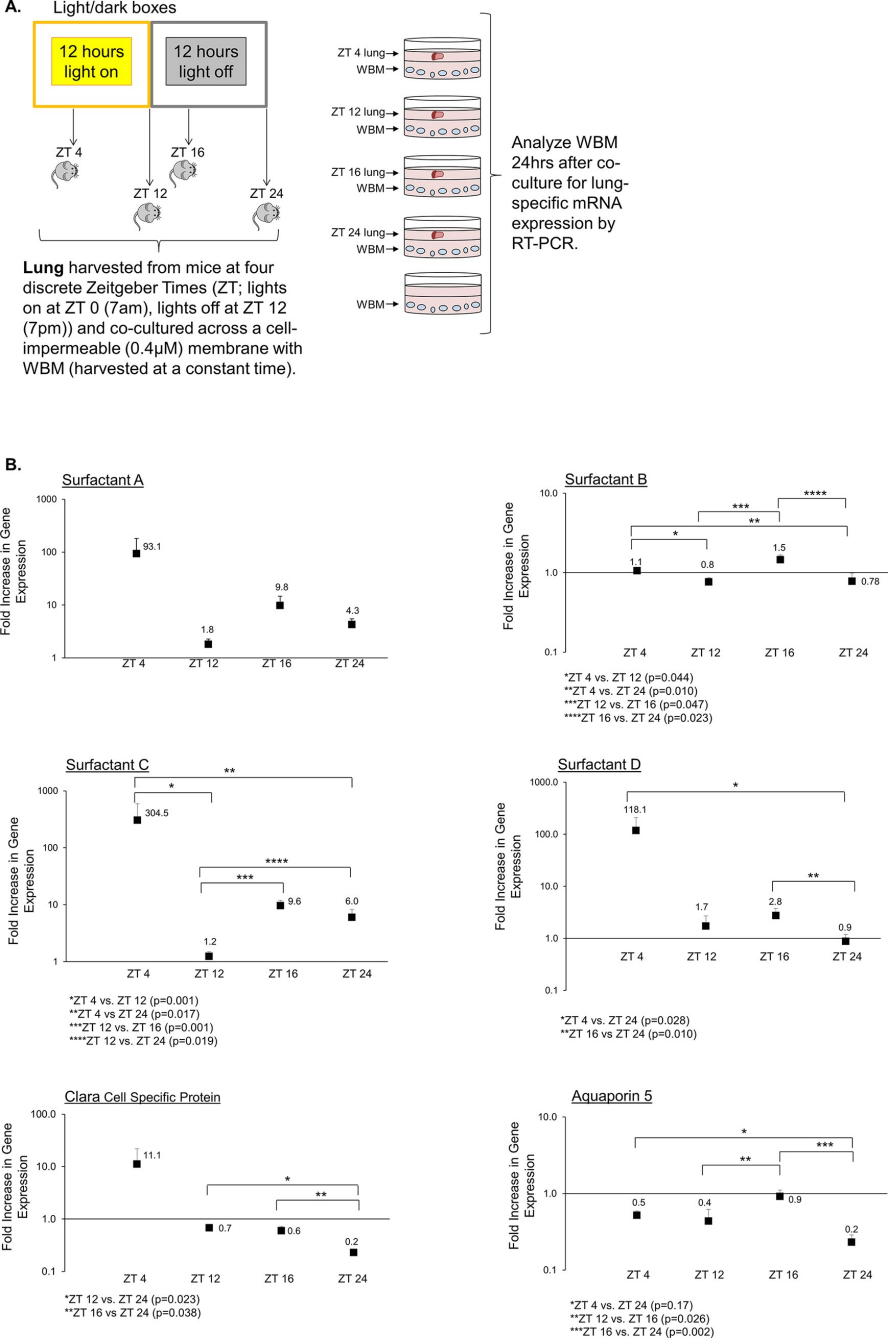
Daily rhythms of the EV donor tissue influence the ability of the EVs to mediate phenotype changes in target WBM. **A.** Methods schematic. **B.** Fluctuations in the levels of multiple pulmonary epithelial genes in EV-exposed WBM were observed when the time of day of EV-donor lung tissue harvest was altered. Target mRNA levels are expressed as fold increase over mRNA levels in WBM cultured in the absence of lung. Data points are average RQ ± sem. **(**Data points represent pooled average expression from n = 12 samples; 2 duplicate wells/mouse x 3 mice/time-point/experiment x 2 experiments). *p<0.05 or as specified in the Figure (Wilcoxon rank-sum test).

As previously published, lung-derived EVs are able to modify the transcriptome of recipient whole bone marrow cells [28–31]. In this in vitro model, WBM cells exposed to lung-derived EVs begin expressing pulmonary-specific mRNAs, providing a quantifiable readout for EV function. We used this well-established model to determine the effects of daily rhythms on the ability of EVs to modulate the phenotype of recipient WBM. First, we addressed whether the time of day for the lung EV donor tissue influenced WBM phenotype modulation. For these experiments, C57BL/6 mice were entrained in 12-hour light/dark boxes. At 4 different ZTs, lungs were co-cultured with WBM, separated by a 0.4μM cell-impermeable well insert. The circadian time-point of harvested WBM cells was kept constant. After 24 hours of co-culture, WBM was collected, RNA was isolated and gene expression for 6 pulmonary epithelial genes was analyzed by RT-PCR (Fig 2A). Fluctuations in the expression of multiple pulmonary epithelial genes in EV-exposed WBM were observed when the daily time-point of the EV-donor lung tissue harvest was altered (Fig 2B). Statistically significant changes were observed in five out of the six pulmonary epithelial genes analyzed (Surfactant B, Surfactant C, Surfactant D, Clara Cell Specific Protein and Aquaporin 5), with highest mRNA levels observed when lung-derived EVs were obtained from mice at ZT 4 and ZT 16. Lowest mRNA levels were seen primarily when the lung-derived EVs were obtained from mice at ZT 12 and ZT 24. There was a trend toward peak mRNA levels at ZT 4 for the other two genes (Surfactant A and Clara Cell Specific Protein), but these differences were not statistically significant.

**Fig 3:**
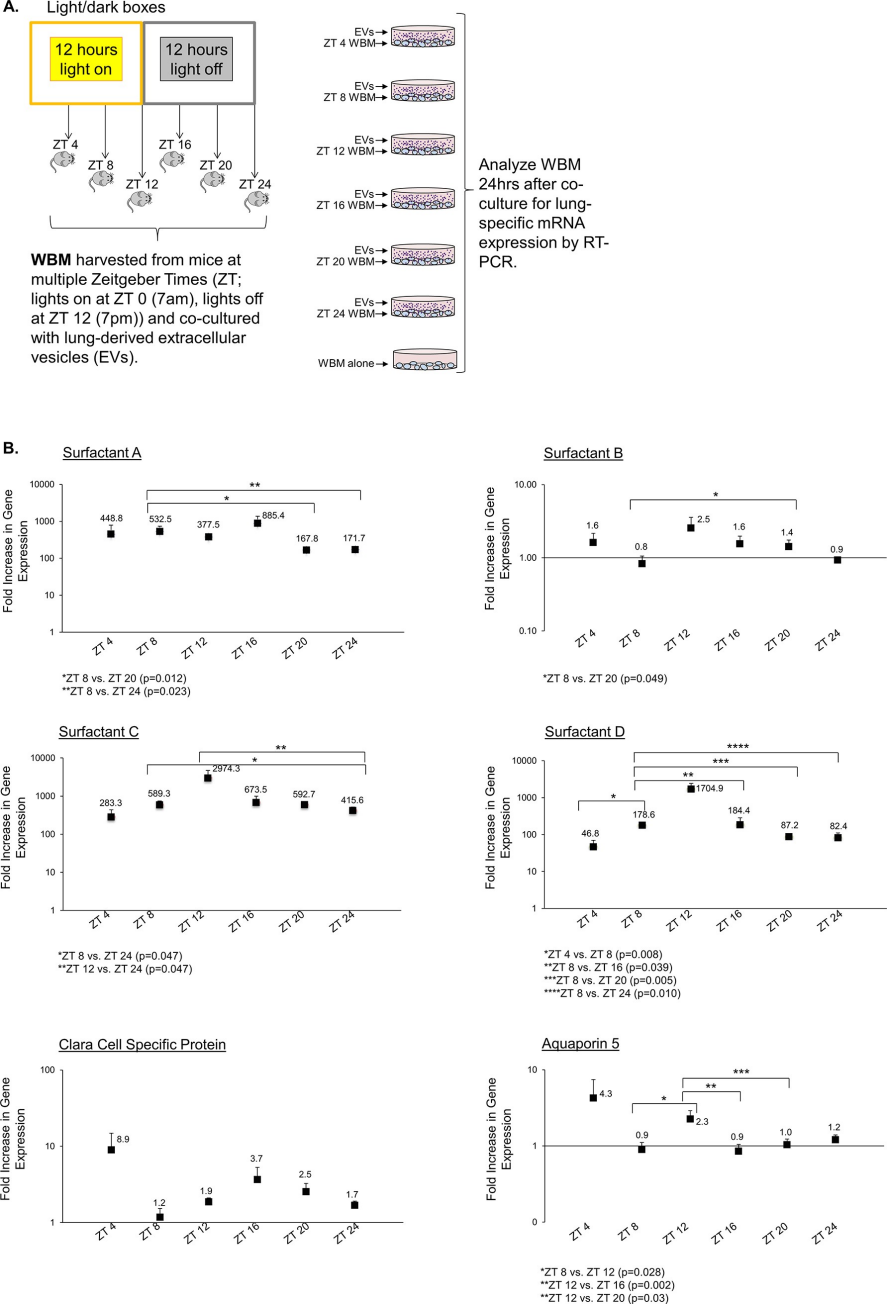
Daily rhythms of target WBM influence the ability of marrow cells to be modulated by lung-derived EVs. **A.** Methods schematic. **B.** Fluctuations in the transcript levels of pulmonary epithelial genes in EV-exposed WBM were observed when the time-point of target WBM cells was altered. Target mRNA levels are expressed as fold increase over transcript levels in WBM cultured in the absence of EVs. Data points are pooled average RQ ± sem representing transcript levels from n = 12 samples, 2 duplicate wells/mouse x 3 mice/time-point/experiment x 2 experiments except for ZT 8 where n = 8 samples. 2 duplicate wells/mouse x 2 mice/time-point/experiment x 2 experiments). *p<0.05 or as specified in the Figure (Wilcoxon rank-sum test).

Second, we altered the time of day for harvesting the recipient WBM cells while keeping the harvest time for donors of lung-derived EVs constant. For this set of experiments, we entrained C57BL/6 mice for 2 weeks. We harvested lungs from entrained mice at ZT 2, and isolated EVs from lung conditioned media using differential ultracentrifugation. We then harvested WBM from entrained mice at 6 different time-points throughout the day (ZT 4, ZT 8, ZT 12, ZT 16, ZT 20, and ZT 24), and co-cultured each time-specific group of marrow cells with lung-derived EVs. After 24 hours in co-culture, we collected WBM, extracted RNA and performed RT-PCR for pulmonary specific epithelial genes (Fig 3A). As shown in Fig 3B, statistically significant alterations in pulmonary epithelial gene mRNA levels were seen when the daily time-point of the recipient WBM cells was altered. Overall, peak pulmonary-specific epithelial gene transcript levels were seen when WBM was harvested at ZT 8–12. The lowest transcript levels were seen when WBM was harvested at ZT 20–24. These data indicate that manipulating only the time of day when vesicle-producing tissues are obtained or when target marrow cells are exposed can dramatically alter this mode of intercellular communication.

**Fig 4:**
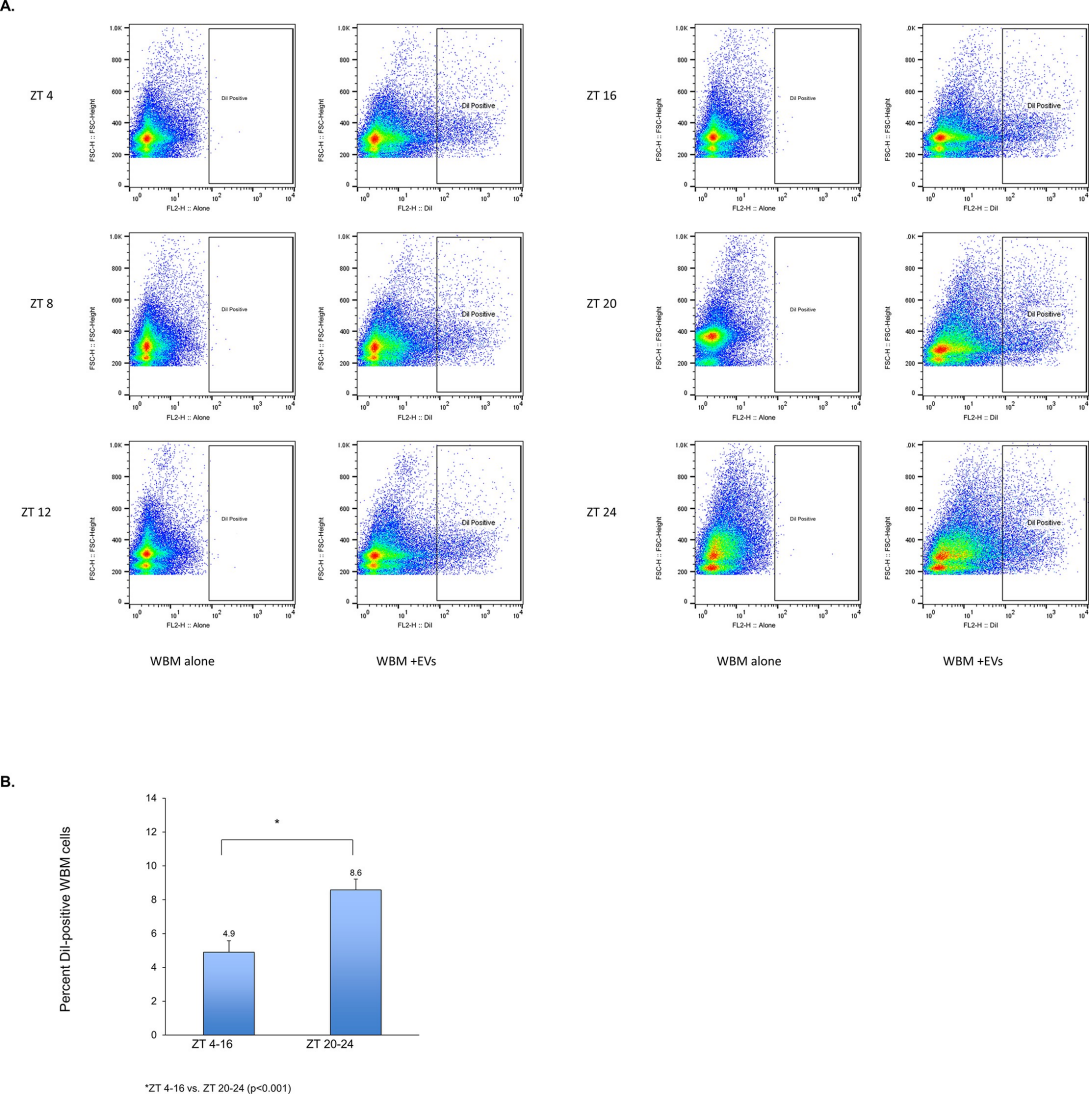
Daily rhythms of WBM cells may influence their susceptibility to EV uptake. **A.** Representative flow cytometric analyses and gating schema. WBM was harvested from mice at ZT 4, ZT 8, ZT 12, ZT 16, ZT 20, and ZT 24. At each discrete time-point, it was incubated with DiI-fluorescently-labeled lung-derived EVs in vitro. After 24 hours in co-culture, WBM was washed and analyzed for percent cells positive for DiI by flow cytometry. For each sample, WBM was gated through FSC/SSC to yield the total cell population, and this population was further gated for DiI positivity. Each sample (WBM + EVs) was run in parallel with WBM cultured in the absence of EVs (WBM alone) as a negative control. **B.** Average percent DiI-positive marrow cells ±standard deviation from 3 individual wells per ZT was calculated (each well = WBM from one mouse). The bars represent pooled average percent DiI-positive marrow cells from ZT 4, 8, 12, 16 and 20 compared to pooled average percent DiI-positive marrow cells from ZT 20 and 24. *p<0.001 (Wilcoxon rank-sum test).

Finally, we tested whether these time of day-dependent changes in pulmonary gene transcript levels were due to differential uptake of EVs. For these experiments, we harvested WBM at 6 distinct time-points throughout the day (ZT 4, ZT 8, ZT 12, ZT 16, ZT 20, and ZT 24), co-cultured the marrow cells with fluorescently-labeled lung-derived EVs for 24 hours, and then determined the percent marrow cell uptake of EVs by flow cytometry (Fig 4). There was a statistically significant, increase in EV uptake or association with WBM cells when the WBM cells were harvested at ZT 20 and ZT 24 when compared to the combined other time-points, suggesting time of day-dependent changes in EV uptake. WBM incubated with DiI processed in the absence of EVs showed no increased fluorescence when compared to WBM alone (data not shown).

## Discussion

EVs have tremendous clinical potential with numerous factors known to alter their biogenesis, release, homing and functionality. To date, there is little known about the effects of circadian rhythm on EVs. For these proof-of-principle studies, we used a model system in which the WBM cell transcriptome is altered by lung-derived EVs. This reliable in vitro read-out for EV function allowed us to begin specifically examining the role of daily rhythms in influencing EV function. The data presented here indicate that daily rhythms at both the donor and the recipient level have the power to influence the ability of EVs to modulate target cell phenotype. For originator lung, there appeared to be two peaks during the day when the lung-derived EVs were best able to increase pulmonary-specific epithelial mRNA in target WBM (ZT 4 and ZT 16) and two nadirs (ZT 12 and ZT 24). For target tissue, WBM was most susceptible to lung-derived EV changes when harvested at ZT 8–12, and less susceptible when harvested at ZT 20–24.

The data presented here describe what we think is an important observation regarding time of day-dependent modulation of EV effects on target tissues, but the mechanisms by which circadian rhythm affect EV biogenesis and function need to be elucidated. When considering mechanisms within the context of these experiments, it is important to note that, although previously published data indicate the induction of target-cell transcription of pulmonary-specific mRNAs by lung-derived EVs [31], in these studies, we are not delineating between mRNA as EV cargo passively transferred to target marrow cells and de novo mRNA transcription within target marrow cells. Therefore, the mechanisms underlying time of day effects could potentially be anywhere from time of day dependent modulation of EV quantity, release, and quality to differential susceptibility of the target tissues with respect to EV uptake, processing and transcriptional changes.

Within our experimental system, we offer the following conclusions regarding possible mechanisms by which time of day influences EV modulation of target cell phenotype. Our data suggest that the time of day-associated peaks and nadirs in pulmonary specific mRNA levels in target WBM are not merely the simple reflection of circadian-related differences in EV quantity or target cell uptake/association. For example, lung tissue EVs were highest in number at ZT 8 and ZT 24 and lowest at ZT 16. If changes in pulmonary-specific mRNA levels in WBM were related only to changes in EV quantity exposure, we would expect peak effects in target WBM when lung was isolated at ZT 8 and lowest when harvested at ZT 16 to mirror lung EV quantity measurements. In contrast, we found nearly the opposite, with the modulation of co-cultured WBM highest when lung was harvested at ZT 16 and lowest at ZT 24. It is important to note that quantification of EVs harvested from lung, marrow and blood at different time-points was done by Nanoparticle Tracking Analysis (NTA) and additional methods to verify the time of day dependent changes in EV quantity we found with NTA will be necessary. With this said, even when the number of lung-derived EVs was kept constant (Fig 3), we still observed fluctuations in the ability of EVs to alter target WBM due to changing the time of day of recipient WBM, again, strongly supporting mechanisms beyond sole time of day dependent changes in EV quantity or quality. Therefore, based on these data, we conclude other time of day-dependent changes in addition to changes in quantity are at play.

Our data suggest there were changes in uptake by target WBM related to daily rhythms of WBM. There was a significant increase in EV association with or uptake by WBM when the WBM was harvested from mice at ZT 20 and ZT 24. In contrast, the peak increase in pulmonary specific epithelial mRNA levels upon EV exposure was when WBM was harvested at ZT 8 with nadirs at ZT 20 and ZT 24. Of note, we are analyzing mRNA levels 24 hours after initial exposure and it may be that the EV uptake at the time of analysis does not necessarily reflect fluctuations in EV uptake over the 24 hour in vitro exposure time. In addition, it may be that EV association with WBM cells as shown by flow cytometry does not always equal EV uptake and processing.

The lack of correlation between time of day-related changes in WBM and changes in EV quantity and target tissue uptake indicates additional mechanistic factors are involved in the influence of daily rhythms on EV function. One mechanism may involve qualitative differences in EV content. Interestingly, studies have shown that levels of rat Surfactant A, B, and C mRNA were higher at 9am and lower at 4pm [32]. Such fluctuations were similar to the trends we observed in WBM co-cultured with lung across cell-impermeable membranes, with elevations in Surfactant B and C expression in target marrow when co-cultured with lung harvested at ZT 4 (11am) and nadirs at ZT 12 (7pm). Therefore, it may be that the EVs at different time-points are enriched for particular pulmonary specific mRNAs based on time of day-related changes in cellular mRNA levels, and that such fluctuations are mirrored in the lung-derived EV cargo. For our studies, we normalized mRNA levels within WBM cells exposed to lung tissue or lung-derived EVs to those levels in un-exposed WBM at each time-point. Therefore, we do not think that our data reflect any potential time of day dependent changes in WBM alone. In addition to possible changes in EV cargo with time of day, Danielson et al, looking at peripheral blood EVs in 6 healthy adults by nano flow cytometry, found fluctuations in size distribution of EVs during the course of a day [25], again, suggesting variations in EV quality related to daily rhythms that may affect their functional capacity. It is likely that numerous changes in EV quality may occur with changes in the time of day to account for increased or decreased function.

With regard to target tissues, daily rhythm-related alterations in target cell susceptibility beyond EV binding/uptake and differential EV cargo likely account for the changes we see in mRNA content in WBM upon EV exposure. This is supported by our data indicating mRNA level changes within target WBM when only the time of day of WBM harvest was altered but the EV input was constant. Interestingly, it has been shown that cell cycle state of the target cell influences its ability to be modulated by EVs. For example, in studies by Aliotta et al, pulmonary-specific mRNA expression in WBM co-cultured with lung across cell impermeable membranes was highest when the target WBM cells were in late G1/early S-phase. Interestingly, if the lung was obtained from irradiated mice, the peak expression was shifted to G0/G1, indicating features of both originator and target tissue govern ultimate EV-mediated effects [33]. Given the clear connections between circadian clocks and cell cycle regulation [34], it is possible that some of the changes we are seeing in pulmonary mRNA levels in WBM exposed to EVs at different time-points throughout the day are related to time of day-dependent cell cycle status changes in the target marrow cells. The daily rhythms involved with vesicle production and function are clearly complicated and vary with induced phenomena investigated (i.e. gene expression or cargo transfer) and originator tissue state.

In these studies, we have demonstrated time of day-dependent changes in the ability of lung-derived EVs to modulate target WBM. Overall, our data indicate that 1) daily rhythm is an important component of EV-mediated inter-cellular communication, 2) the daily rhythm of both the originator tissue and of the target cell influences the biologic effects of EVs, and 3) these functional effects may not be directly correlated with simple changes in EV quantity exposure or differential uptake. It is unknown how circadian oscillations exert their effects on EV-mediated communication, how such effects will vary with different originator and target tissues and in what ways these effects will alter EVs in different functional contexts. Additionally, in the experiments presented here, we controlled for light/dark cycles but myriad other phenomena including time of day related changes in activity and diet might certainly be playing a role in EV-mediated intercellular communication. Elucidation of the mechanisms by which such time of day dependent oscillations modulate EV function will be important to help guide more successful EV manipulations in the future.
